# A Study of the Frequency of Lead Reversal at a Tertiary Care Institution

**DOI:** 10.7759/cureus.92345

**Published:** 2025-09-15

**Authors:** Srinivasan Ramadurai, Visvarath Varadarajan, Alwyn Alec Lasrado, Bharath Vignesh R K, Ramya Venkatesan, Sowmya Gopalan

**Affiliations:** 1 General Medicine, Sri Ramachandra Institute of Higher Education and Research, Chennai, IND

**Keywords:** 12-lead ecg, ecg (electrocardiogram), lead reversal, limb lead reversal, precordial lead reversal

## Abstract

Background: Electrocardiography (ECG) remains an important point-of-care investigative modality in the screening and diagnosis of cardiac diseases due to rapidity, cost-efficiency, and a gentle learning curve for most emergent patterns. Mechanistical problems in recording ECGs can lead to distorted graphical representations of cardiac electrophysiology, which can lead to incorrect or obstructed care for the patient.

Aim: To identify the prevalence of ECG lead reversal in a tertiary care hospital over two months and to discuss the various patterns of ECG lead misplacement.

Methods: A total of 1,000 ECGs were screened over a period of two months. ECGs with features suspicious for lead misplacement were identified, the corresponding patients had a repeat ECG taken under supervision, and the presence or absence of lead misplacement was verified on the basis of chest lead progression, inverted limb complexes, and isolated flatlines. ECGs of paediatric patients (<18 years) and patients with cardiac axis abnormalities (e.g., dextrocardia) were excluded.

Results: Fifteen out of 1,000 ECGs were confirmed to have lead misplacement (1.5%). Eleven (1.1%) of these had limb lead reversal, of which eight were LA-LL reversal (0.8%) and three were RA-LA reversal (0.3%). Four ECGs had evidence of chest lead reversal (0.4%).

Conclusion: It is important for healthcare professionals to learn to recognize electrical patterns of lead reversal, as the ECG must be retaken promptly and carefully after repositioning the leads correctly, in order to avoid delays or disturbances in the care of patients.

## Introduction

In the modern era of diagnostics, electrocardiograms (ECGs) remain an important and extensively used diagnostic modality across emergency rooms (ERs), intensive care units (ICUs), and general wards in the evaluation of arrhythmias, myocardial infarction, conduction blocks, and many other cardiac conditions. Despite its importance, errors in ECG acquisition, including lead misplacement, are not uncommon and can lead to significant misinterpretation [1-3].

Care must be taken in the precise positioning of ECG leads and electrodes, as each lead is interpreted in the context of a specific region of the heart. When leads are exchanged or misplaced, the electrical activity is incorrectly recorded and represented, which can change the management of the patient. Globally, the incidence of ECG lead misplacement ranges from 0.4% to 4%, as per a study performed in 2007 [4]. More relevant and recent data regarding the prevalence of technical problems in obtaining ECGs is lacking. Moreover, these errors are often under-reported and can result in critical clinical consequences if not recognised in time. The most frequent type of misplacement, as per the literature, involves reversal of the right arm (RA) and left arm (LA) leads. Such misplacement can mimic life-threatening conditions such as inferior wall myocardial infarction or Brugada syndrome, leading to inappropriate clinical decisions [5-7]. This may at best generate unnecessary panic, and at worst lead to the administration of treatments that do not correspond to the actual physiological or pathological state of the patient. Awareness among junior doctors, nurses, and paramedical staff regarding the identification of such patterns is often lacking. Hence, this study was undertaken to systematically analyse the patterns of lead misplacement in a tertiary care setting.

In this study, we will aim to determine the rate of occurrence and types of ECG lead misplacements seen in a tertiary care hospital. We will also describe the ECG patterns associated with different configurations of lead misplacements.

## Materials and methods

This cross-sectional study was conducted over a period of two months, with the aim of identifying the frequency of lead misplacement occurring while obtaining ECGs in our tertiary care centre. The study was approved by the Institutional Ethics Committee of Sri Ramachandra Institute of Higher Education and Research (IEC-NI/21/APR/78/81). Consent was obtained for all study participants before the collection of data.

The ECGs of all consenting adult patients (age greater than or equal to 18 years) who presented to the ER or who had been admitted to the wards or into the ICU during the study period were included in the study. All ECGs were screened for signs suspicious for lead misplacement (e.g., inverted P, QRS, or T waves in limb leads, flat lines in the limb leads, or non-uniform progression of R waves in the chest leads). For patients who had such ECG findings, one repeat ECG was taken under supervision after ensuring all the leads were correctly placed. The old and repeated ECGs were compared by three general medicine consultants (of whom two are senior consultants) and one cardiologist, and the presence or absence of lead misplacement was decided by at least three concurring opinions. When an ECG received an equal number of opinions indicating lead misplacement and opinions against lead misplacement, the status of the ECG was decided based on discussion and consensus. Patients were managed according to their clinical presentation as per guidelines, with repeated ECG under consideration.

Excluded were ECGs of paediatric patients (age less than 18 years). Among adult patients, exclusion criteria included nonconsent, ECGs of patients with known or later identified physical cardiac axis abnormalities such as dextrocardia, and ECGs with excessive artefact or poor quality that could not be read satisfactorily. A total of 143 adult ECGs were excluded this way.

Given the prevalence of 4% in earlier literature [4], with a precision of 1.5% and a confidence of 95%, the ideal sample size for adequate study power was calculated as 657. Accounting for a 25% non-consent rate, the final required sample size was calculated to be 820. For convenience, a sample size of 1,000 was chosen. The frequency of lead misplacement noted was represented using a percentage point prevalence with a Clopper-Pearson 95% confidence interval. Statistical analysis was performed using R software (version 4.5.1; R Foundation for Statistical Computing, Vienna, Austria) using the 'binom' package for data analysis and ‘ggplot2’ package for the generation of graphs.

## Results

In this cross-sectional observational study, the presence of lead misplacement in the 1,000 ECGs accepted was reviewed. In this sample of 1,000, the exact frequencies of verified lead exchange and patterns of misplacement noted were as follows (Table 1).

**Table 1:** Breakup of patterns of ECG lead reversal. Two patterns of limb lead reversal and four patterns of chest lead reversal were identified. LA, left arm electrode; LL, left leg electrode; RA, right arm electrode

Value	Number	Study Prevalence (95% Confidence Interval)
Number of ECGs screened	1000	
Number of ECGs with confirmed lead reversal	15	1.5% (0.84, 2.46)
Number of ECGs with limb lead reversal	11	1.1% (0.55, 1.96)
Patterns of limb lead reversal		
LA–LL reversal	8	0.8% (0.35, 1.57)
RA–LA reversal	3	0.3% (0.06, 0.87)
Number of ECGs with chest lead misplacement	4	0.4% (0.11, 1.02)
Patterns of chest lead misplacement		
V2–V3 exchange	1	0.1% (0.003, 0.56)
V2–V4 exchange	1	0.1% (0.003, 0.56)
V3–V5 exchange	1	0.1% (0.003, 0.56)
V2–V5 shuffling	1	0.1% (0.003, 0.56)

Of the flagged 1,000 ECGs, 15 of them (1.5%) showed continued evidence of lead misplacement when the ECG was repeated. Of these 15 ECGs, 11 (1.1%) were due to limb lead reversal, and four (0.4%) were due to chest lead reversal. Two major patterns limb lead reversal patterns emerged: eight cases (0.8%) featured left arm electrode (LA)-left leg electrode (LL) exchange that was recognised by negative P, QRS, and T waves in lead III; and three cases (0.3%) featured right arm electrode (RA)-LA exchange recognised by negative P, QRS, and T waves in lead I and aVL.

Of the four chest lead misplacement cases, one case involved exchange of V2 and V3; one case involved exchange of V2-V4 (with V2 placed over V3's location, V3 over V4's location, and V4 placed over V2's location); one case involved exchange of V3-V5 (with V3 placed over V4's location, V4 placed over V5's location, and V5 placed over V3's location); and one case involved exchange of V2-V5 (with V2 placed over V3's location, V3 placed over V4's location, V4 placed over V5's location, and V5 placed over V2's location). In our study, we did not find any ECGs with evidence of inappropriate placement of chest leads (e.g., high V1/V2).

The p-value for the difference between the prevalence of limb lead reversals and the prevalence of chest lead reversals in our study, as calculated by the two-tailed z-test, was 0.0035. The prevalence of limb lead reversals is therefore statistically significantly higher than the prevalence of chest lead reversals. In view of there being four observers, interobserver agreement was calculated using Fleiss' kappa coefficient, which was 0.731 (substantial agreement) for all 1,000 datapoints (p <0.001).

**Figure 1:**
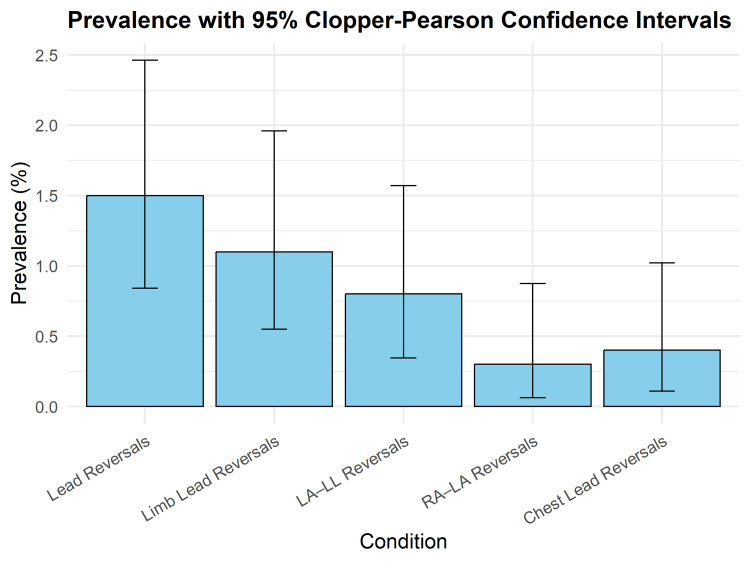
Bar plot representing study prevalences, with error bars representing Clopper-Pearson 95% confidence intervals for the categories of lead reversal presented in Table 1. Image credit: Dr Srinivasan Ramadurai. Graph generated by R software using the ggplot2 module.

A graphical representation of the prevalences presented in Table 1 is as follows (Figure 1).

**Figure 2:**
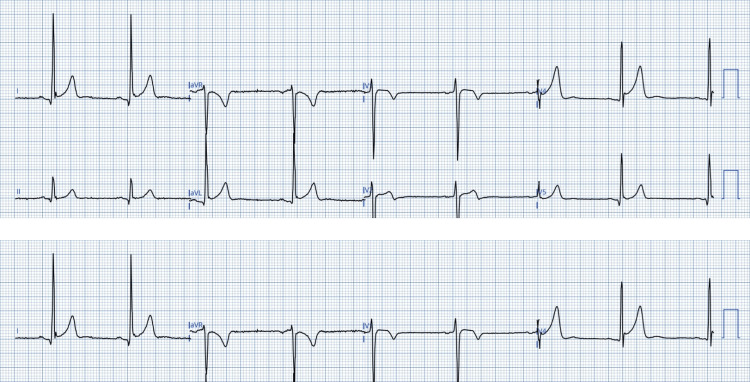
ECG demonstrating LA/LL limb lead reversal. Patient ECG digitsed and anonymised using PMCardio app. Image credit: Dr Srinivasan Ramadurai

Example ECGs of study participants are shown below. Figure 2 shows the ECG of a study participant exemplifying LA/LL lead reversal. The primary finding is that of isolated lead III P, QRS, and T wave inversions, while aVR remains physiologically negative. More subtly, the leads I and II have switched, so have the leads marked aVL and aVF.

**Figure 3:**
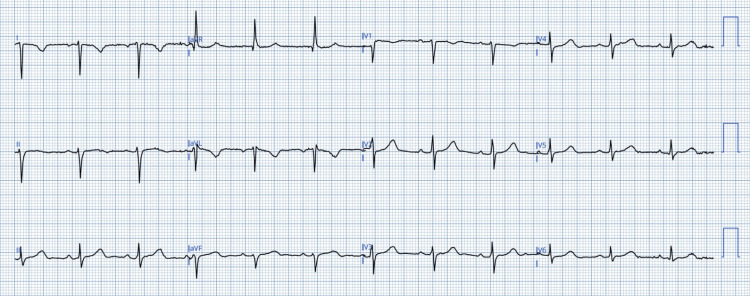
ECG demonstrating LA/LL limb lead reversal. Patient ECG digitsed and anonymised using PMCardio app. Image credit: Dr Srinivasan Ramadurai

Figure 3 shows the ECG of a study participant exemplifying RA/LA lead reversal. Here, there is a positive inversion of the waves in aVR; this is because the lead marked aVR actually depicts aVL. Additionally, lead III has inverted.

## Discussion

The overall prevalence of lead reversal during the observed study period was 1.5% (95% CI: 0.84-2.46%). Though the overall frequency of reversal events in our study was low, this prevalence could be clinically impactful when considering the implications of an incorrectly read ECG. As mentioned earlier, this could lead to the initiation of incorrect treatment for the patient, which could be unnecessary at best and actively harmful at worst. This is consistent with the findings of previous studies that provide a prevalence of 0.4-4% [4].

Lead reversal or misplacement is not very uncommon in hospital settings. It is frequently encountered in areas of urgency and rapid action sequencing, such as the emergency room or the critical care units [2,3]. Misplacements or reversals occur both in limb leads and chest leads. Normally, misplacements do not occur between limb leads and chest leads because the limb leads are color-coded and are made as clamps to attach onto the corresponding limbs, whereas the chest leads are usually in the form of rubber bulbs or pads attached to the chest wall and are colored differently from the limb leads [8].

Misplacement of Limb Leads

There are four limb leads corresponding to the four limbs to which they are attached, namely the right arm (RA), left arm (LA), left leg (LL), right leg (RL or N for neutral). Indian ECG machines follow the colour-coding recommendations set by the International Electrotechnical Commission (IEC) [9], per which the RA electrode is depicted as red, the LA electrode as yellow, the LL electrode as green, and the RL/N electrode as black.

**Figure 4:**
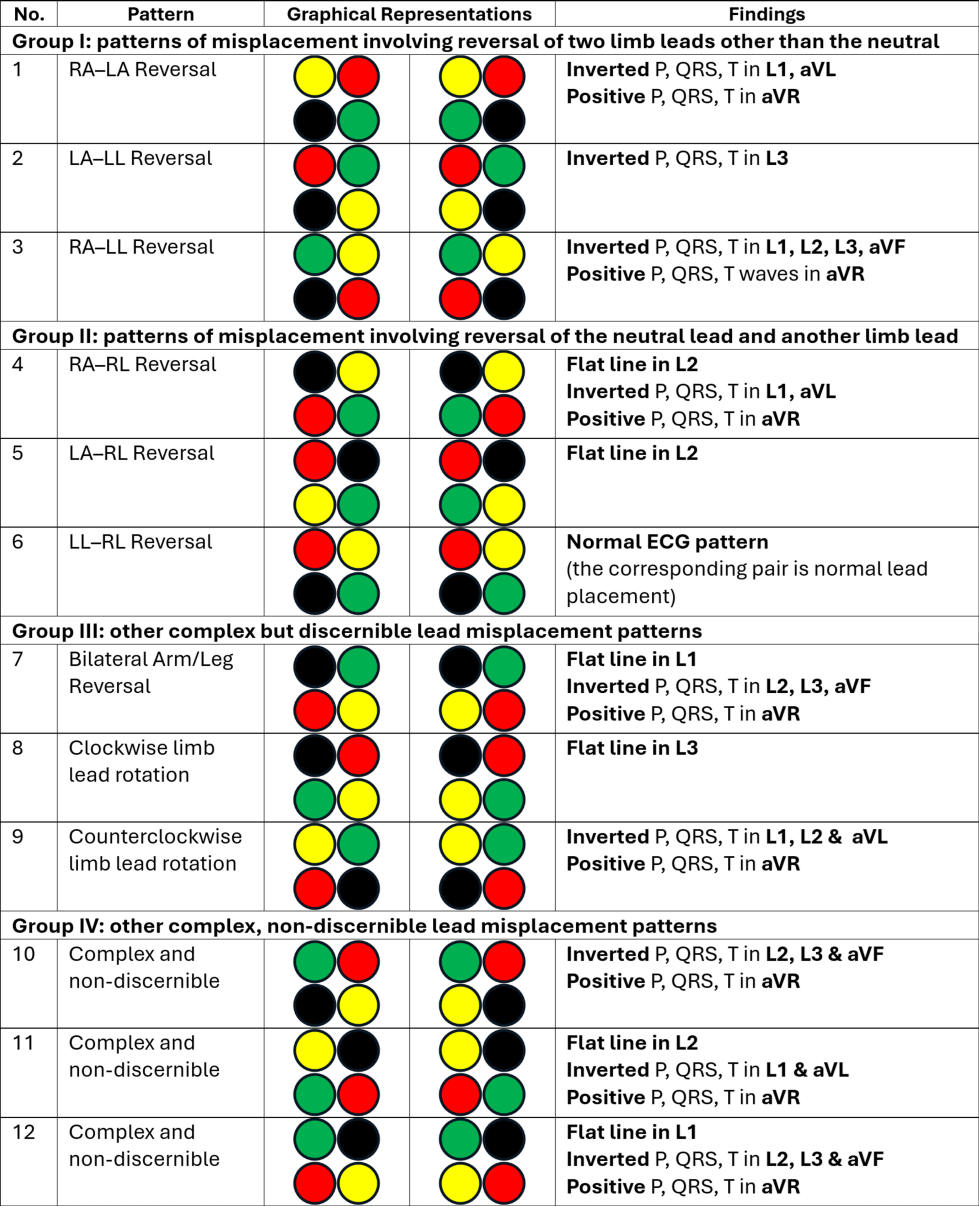
Graphical representation of limb lead reversal patterns. 1-3: Three discernible patterns of reversal of two limb leads without involvement of the neutral lead. 4-6: Three discernible patterns of reversal of two limb leads, of which one is the neutral lead. 7-9: Three other discernible lead misplacement patterns involving the complex exchange of multiple leads. 10-12: Three difficult-to-discern lead misplacement patterns involving the complex exchange of multiple leads. As per IEC conventions, left arm (LA) is represented by a yellow circle, right arm (RA) by a red circle, left leg (LL) by a green circle, and right leg (RL) by a black circle. Image credit: Dr Srinivasan Ramadurai

There are 24 possible combinations of lead placement with these four limb leads (Figure 4). As interchange of the electrodes placed on the lower limbs does not generally greatly disturb the directions of the leads recording electrocardiac activity, these 24 combinations can be paired into 12 pairs. Of these 12 pairs, there are nine easily discernible patterns. The first group of three pairs of patterns is of lead reversal without involvement of the neutral lead (RA-LA reversal, RA-LL reversal, and LA-LL reversal) [10-13]. The next group of three pairs is of lead reversal involving the neutral lead (RA-RL reversal, LA-RL reversal, and LL-RL reversal) [11,14,15]; one of these patterns is actually the normal placement of the limb leads (the leg-lead reversed variation of LL-RL reversal). The remaining group of three pairs is of other complex discernible patterns (bilateral arm lead reversal with leg lead reversal, clockwise rotation of leads, and counterclockwise rotation of leads) [10-12]. The last three pairs of patterns include complex misplacements of multiple leads whose exact configurations are difficult to discern; although the presence of lead misplacement can be identified by the two broad electrical patterns that emerge, complete reversal of P, QRS, and T waves in one or more leads and flat line in any of the limb leads.

The combinations that are possible, other than those described above, are complex and involve more than two lead misplacements. The exact leads misplaced in these ECGs are tough to identify. All of them will have one lead with a flat line or two or more leads with inverted P, QRS, and T waves.

**Figure 5:**
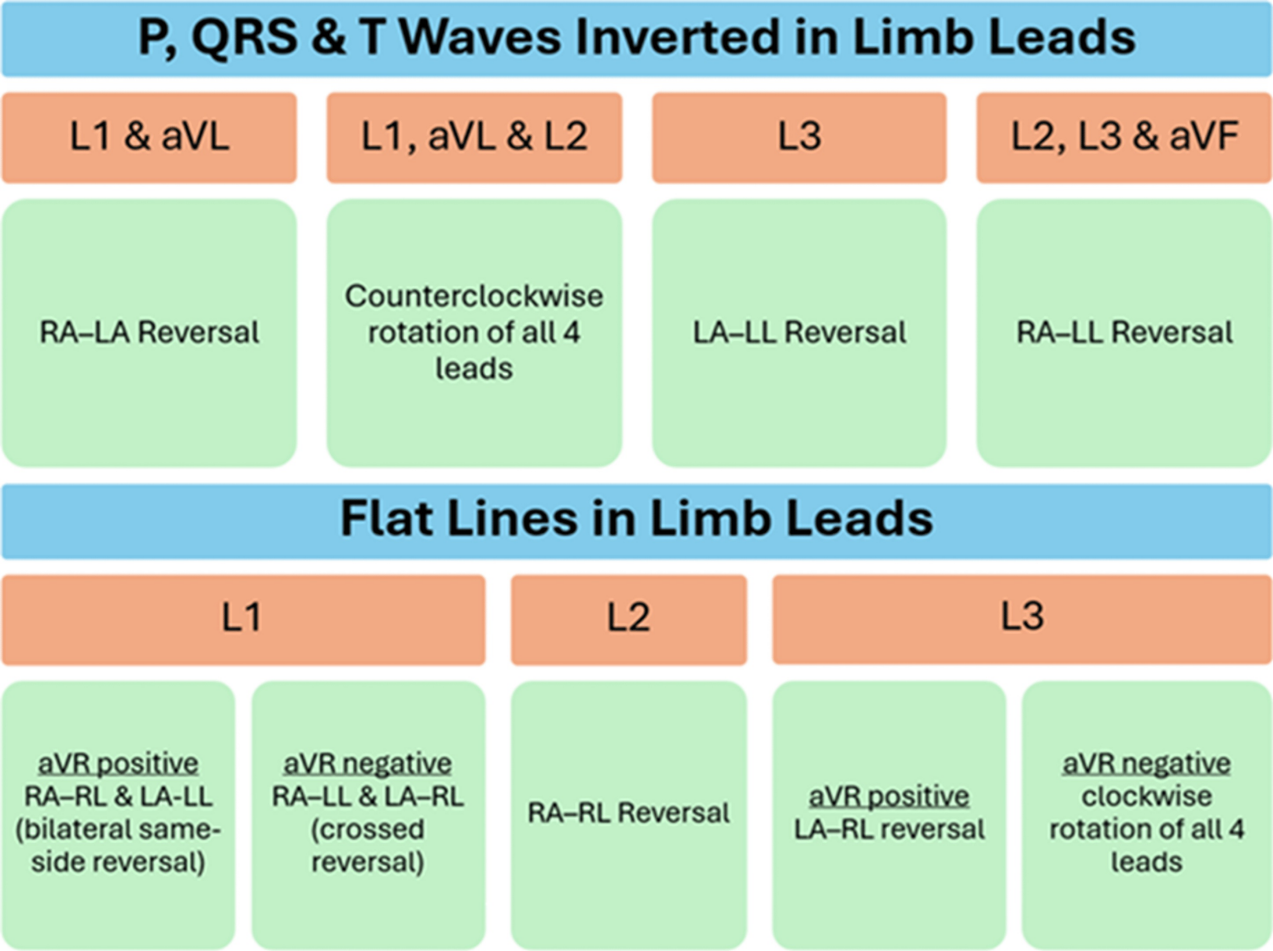
Diagnostic approach to the presence of P, QRS, and T wave inversion in limb leads and flat lines in limb leads. Image credit: Dr Srinivasan Ramadurai LA: left arm (LA); RA: right arm; LL: left leg; RL: right leg

Figure 5 presents a graphical summarization of the approach towards suspected limb lead reversal.

Misplacement of Chest Leads

**Figure 6:**
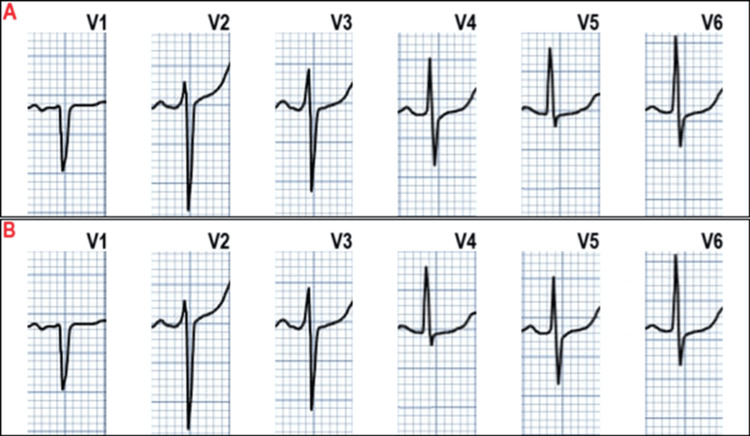
Pane A. Normal progression of R wave morphology in the chest leads. Pane B. Abnormal progression of R wave morphology in the chest leads, implying chest lead reversal. Image credit: Dr Srinivasan Ramadurai. ECG digitised with the PMCardio app.

Misplacement of chest leads is quite common [16-18]. The leads are rubber bulbs of uniform color, numbered from V1 to V6. It is common for adjacent leads to be reversed. The normal progression of R waves from V1 to V6 (Figure 6A) is disrupted by exchange or misplacement of the chest leads (e.g., Figure 6B).

The next most common type of chest lead misplacement is when leads are not placed in the appropriate intercostal spaces. Per recommendations, V1 is placed in the right fourth intercostal space near the sternum, V2 in the left fourth intercostal space near the sternum, V4 in the left fifth intercostal space in the midclavicular line, V3 in between V2 and V4, V5 in the left fifth intercostal space in the anterior axillary line and V6 in the left fifth intercostal space in the midaxillary line [18,19]. Placement of chest leads outside of these positions can lead to false patterns; for example, when V1 and V2 are placed in the right and left second intercostal space instead of the fourth, the ST segment will be abnormally elevated, mimicking myocardial infarction or a Brugada pattern [5-7,19,20].

It is therefore quite important to encourage vigilance among trainees to suspect lead exchange or misplacements, especially when the ECG pattern is critical, unusual, or non-correlative with the clinical gestalt of the patient. Recommendations for accurate lead placement and interpretation must constantly be kept in mind [21-23]. Electrophysiological patterns that should raise suspicion for lead reversal have been presented in a simple manner in this discussion. Most major centres use ECG machines that provide a machine reading of the ECG as well, but this is often erroneous; in our setting, the machine was unable to recognise lead misplacement in any of the ECGs that human consensus determined showed features of lead misplacement. Most popular modern ECG rhythm analysis software programs also do not often account for the possibility of misplaced leads in their analysis, often reporting signs of reduced ejection fraction or ST-elevation myocardial infarction erroneously. While improvements in machine learning software could certainly increase their value as an ancillary tool in the identification of mechanistic problems such as lead reversal, these are not a total replacement for human expertise. To that extent, a better education on lead misplacements and how to identify them must be given to physicians, nurses, technicians, and paramedical staff. For healthcare professionals for whom the detailed interpretation of ECGs is not a primary responsibility, the introduction of ECG reading checklists or algorithms, especially printed checklists, might assist in reducing interpretational errors, reducing the incidence of lead reversal by encouraging repeat ECG, and improving the rates of rapid escalation of problematic ECGs.

Study limitations

The study period is narrow, leading to a smaller sample size. This also limits the ability to analyse the exact frequencies of each type of limb lead reversal or chest lead reversal, limiting the precision of the prevalence estimates and widening confidence intervals. Multicentric studies with longer study durations and larger sample sizes are needed to be able to examine the rates of different types of discernible or non-discernible lead misplacements. Additionally, ECGs could be stratified based on the urgency with which they were taken (in high-urgency settings, such as in ERs or during emergencies, versus in more relaxed scenarios, such as when taken routinely, for monitoring, or for diagnosis of less urgent conditions); the likelihood of misplacement of leads in either scenario may also be examined this way. As the prevalence of lead reversal is low, a tighter precision would be more beneficial in improving the external validity of the study.

## Conclusions

The prevalence of 1.5% in our study shows that ECG lead misplacements can still occur when care is not taken, despite the current standards of ECG education. Limb lead misplacement is more common than chest lead misplacement. Limb lead misplacement may be suspected when there is the presence of either complete inversion of P, QRS, and T or a flat line in any of the limb leads. Lead misplacements involving the chest leads disrupt the normal progression of R waves. Abnormal placement of leads in the inappropriate areas can also result in erroneous diagnoses, which can be prevented by early recognition and prompt repeat ECG. Incorporation of lead reversal screening into ECG assessment algorithms and introduction of formal, standardised ECG interpretation education for all levels of healthcare professionals can substantially mitigate risks associated with improper ECG technique or reading.
